# Drosophila: Retrotransposons Making up Telomeres

**DOI:** 10.3390/v9070192

**Published:** 2017-07-19

**Authors:** Elena Casacuberta

**Affiliations:** Institute of Evolutionary Biology, IBE, CSIC—Pompeu Fabra University, Barcelona Spain, Passeig de la Barceloneta 37-49, 08003 Barcelona, Spain; elena.casacuberta@ibe.upf-csic.es; Tel.: +34-932309637; Fax: +34-932309555

**Keywords:** telomere integration, *HeT-A*, *TART*, *TAHRE*, Drosophila, telomere targeting

## Abstract

Drosophila and extant species are the best-studied telomerase exception. In this organism, telomere elongation is coupled with targeted retrotransposition of *Healing Transposon* (*HeT-A*) and *Telomere Associated Retrotransposon* (*TART*) with sporadic additions of *Telomere Associated and HeT-A Related* (*TAHRE*), all three specialized non-Long Terminal Repeat (non-LTR) retrotransposons. These three very special retroelements transpose in head to tail arrays, always in the same orientation at the end of the chromosomes but never in interior locations. Apparently, retrotransposon and telomerase telomeres might seem very different, but a detailed view of their mechanisms reveals similarities explaining how the loss of telomerase in a Drosophila ancestor could successfully have been replaced by the telomere retrotransposons. In this review, we will discover that although *HeT-A*, *TART*, and *TAHRE* are still the only examples to date where their targeted transposition is perfectly tamed into the telomere biology of Drosophila, there are other examples of retrotransposons that manage to successfully integrate inside and at the end of telomeres. Because the aim of this special issue is viral integration at telomeres, understanding the base of the telomerase exceptions will help to obtain clues on similar strategies that mobile elements and viruses could have acquired in order to ensure their survival in the host genome.

## 1. Introduction

Early work by H.J. Muller [1] showed that the end of the chromosome cannot be simply a blunt end, and must involve a specialized structure. Later, Barbara McClintock demonstrated that this was actually true and, if left unprotected, the ends of chromosomes could fuse to each other and enter in a “breakage-fusion cycle” with deleterious consequences for the cell [2]. So, it was already long ago that it was established that the ends of the chromosomes need something that distinguish and protect them in particular. In addition, the incapacity of the cellular DNA polymerases to move in a 3′ to 5′ direction opened the need for a special elongation mechanism for the telomeres [3]. The discovery of a specialized polymerase, telomerase, that carries its own RNA template and is in charge of telomere elongation in most eukaryotes, seems to complete the picture for eukaryote telomeres [3].

Drosophila telomeres were the first to be named as such, but life is full of surprises and Muller’s first-described telomeres would turn out to be an exception to the otherwise highly-conserved eukaryote telomeres [4]. Drosophila telomeres are composed of repeated sequences like its telomerase counterparts, but in this case, the repeats expand from six to twelve kilobases (kb) in length and have a complex internal structure. Each repeat contains promoters, genes, and regulatory sequences involved in their own maintenance [5,6,7]. The telomeres in Drosophila are constituted exclusively by head-to-tail arrays, always in the same direction, of randomly transposed copies of three specialized non-Long Terminal Repeat (non-LTR) retrotransposons, *Healing Transposon* (*HeT-A*), *Telomere Associated Retrotransposon* (*TART*) and *Telomere Associated and HeT-A Related* (*TAHRE*) [8,9].

Far from being relegated to a rare exception, Drosophila telomeres have been the object of intense study in the past twenty years [6,9,10,11]. It was and still is intriguing to understand how transposable elements (TEs), whose nature is mainly parasitic, and which are apparently unrelated to telomerase, are able to fulfill an essential cellular function. Moreover, from an evolutionary perspective, it is puzzling to understand how the telomeric transposons were efficiently integrated into the diverse features and pathway interactions related to telomere biology. Studies from many laboratories focusing on different aspects have been slowly piecing together a picture that cannot only describe this alternative mechanism in detail, but also enlighten telomere, transposon, and genome biology in general (see [11] for the latest review).

Regarding this special issue of Viruses, we should keep in mind how studies in Drosophila as well as in other telomerase exceptions, are suggesting that although the telomere sequence is quite well conserved in eukaryote evolution, some essential features of telomere biology such as end protection and stability (telomere capping) are in fact sequence independent [12,13,14]. This phenomenon allows, therefore, an unexpected plasticity to those sequences occupying this genome location. In addition, the study of the similarities between telomerase and retrotransposons has brought to light the mechanistic connections that are also supported from an evolutionary point of view [4]. Because TEs and viral genomes keep structural and evolutionary similarities, it is not unreasonable to consider possible TE strategies for telomere and genome integration in general to obtain clues to better understand viral integration at telomeres.

Because this review on Drosophila telomeres is integrated in this special issue of viral integration at telomeres, I will concentrate in those aspects of telomere biology in Drosophila which I believe might be more relevant to the above-mentioned question. Therefore, I advise those readers looking for a wide review on the latest aspects of telomere biology in Drosophila that although many aspects of what is known about telomere biology in Drosophila have been here put together, this won’t be a work as complete as others already available in the literature [11,14].

## 2. Telomere Transposition

### 2.1. The Players

**Figure 1:**
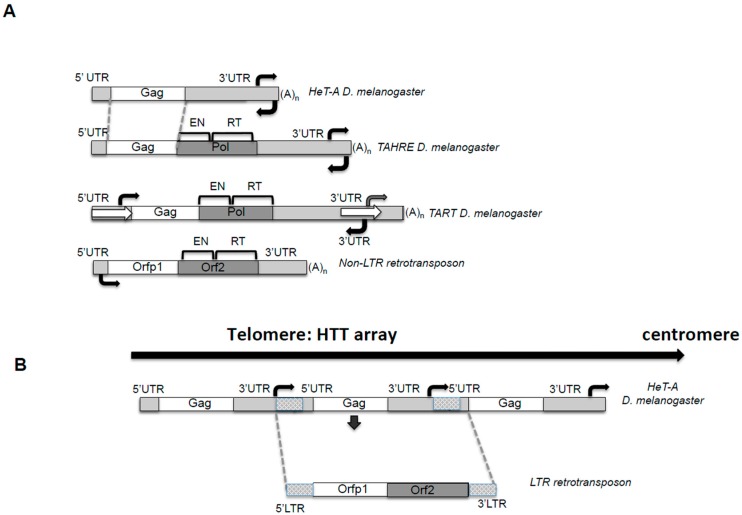
The telomeric retrotransposons of Drosophila. (**A**) The telomere retrotransposons. Figure 1 depicts the telomeric retrotransposons *Healing Transposon* (*HeT-A*), *Telomere Associated and HeT-A Related* (*TAHRE*) and *Telomere Associated Retrotransposon* (*TART*) from *Drosophila melanogaster* and a canonical non-Long Terminal Repeat (non-LTR) retrotransposon for comparing the unusual features of the telomere retrotransposons. Figure 1 is drawn approximately to scale. Dotted grey lines show conserved regions of *TAHRE* and *HeT-A* DNA sequences. Bright Grey: non-coding 5′ and 3′ untranslated regions (UTRs) sequences. White: Gag open reading frames (ORF). Dark Grey: Pol ORF domains; EN, endonuclease, RT, reverse-transcriptase. White arrows in *TART* indicate the Perfect Non-Terminal Repeats (PNTRs); (A)n, 3′ oligo A. Black Arrows: indicate approximate location of the sense and antisense promoter. (**B**) The *HeT-A* telomere retrotransposon resembles an intermediate between a non-LTR and a LTR retrotransposon. Representation of a *Drosophila melanogaster* telomere. The array of *HeT-A* elements shows how from sense to sense promoter its analogous to an LTR retrotransposon. See legend in A) for schematic representation.

Drosophila telomeres are constituted by mainly two kinds of repeats, *HeT-A* and *TART*, which expand, on average, from six to fourteen kb in length [15]. These repeats not only encode for the enzymatic activities needed to elongate the telomere by transposition, but also for other proteins that will be involved in telomeric targeting and for the regulatory sequences involved in the control of their transcription and transposition [6,16]. Telomere repeats in Drosophila are complete genetic units and they correspond to multiple copies of three different non-LTR retrotransposons, *HeT-A*, *TART*, and *TAHRE*, from now on “*HTT* array” [6,8,17] (Figure 1).

Retrotransposons belong to Class I transposable elements, and their mechanism of transposition involves an RNA intermediate, implying that each new successful transposition will result in an increased number of copies of the element [18]. From this point of view, having a retroelement copying itself exclusively onto the end of the chromosome when needed is a beneficial mechanism for a genome that lost the enzyme in charge of this function. *HeT-A*, *TART*, and *TAHRE* are non-LTR retrotransposons [8,19] (Figure 1A). It is important to understand which features of these TEs are common to their counterparts that insert in other genomic locations, and which might be an adaptation to the telomeric role. Certainty, the HTT array shows some unusual features that are conserved across Drosophila species [20].

In Figure 1, I have drawn the structure of a canonical non-LTR retrotransposon by the current definition [18]. Comparing this consensus structure with the one of the *HTT* elements, the commonalities are revealed. The elements have 5′ and 3′ untranslated regions (UTRs), which contain promoter and regulatory sequences, an end with a poly A tail, and encode for two open reading frames (Orf), Orfp1, with structural functions, and Orf2 or Pol, with enzymatic activities [18]. Next, I will describe in detail some features of the *HTT* elements that deviate from this canonical description.

#### 2.1.1. The UTRs and the Bidirectional Transcription

*HeT-A*, *TART* and *TAHRE* contain an apparently standard 5′UTR and an unusually long 3′UTR (Figure 1A). In the case of *HeT-A* and *TAHRE*, both 5′ and 3′UTRs contain sequences working as sense promoters. Sequential deletions from the entire 3′ and 5′UTR were used to define the strongest promoter of the element [4,21]. This apparently shocking combination makes complete sense if, instead of looking at the individual TEs, one pictures the telomere sequence in Drosophila. The *HTT* elements are organized in tandem head-to-tail arrays always in the same direction, and therefore, sequences of the 3′ of one element are followed by the 5′UTR of the element immediately downstream Figure 1B. Interestingly, this solution would also efficiently buffer the possible 5′UTR erosion from being at the end of the chromosome, protecting the element from the possible loss of its promoter. Actually, if the element is considered as a genetic unit from promoter to promoter, the structure resembles that of an LTR retrotransposon, (Figure 1B) suggesting a possible evolutionary relationship of *HeT-A* and *TAHRE* with LTR-retrotransposons [21].

The three *HTT* elements bear antisense promoters in their UTR sequences. *TART* elements in all species are expressed in both sense and antisense orientations, and in some species the antisense transcription is much more abundant than the sense orientation [20,22]. *HeT-A* and *TAHRE* are expressed mainly in sense orientation but antisense transcripts have also been detected, revealing the presence of antisense promoters [5,6,23,24]. Importantly, a detailed study on *HeT-A* antisense transcription revealed the presence of conserved spliced variants [25]. The fact that most orthologues of the telomere retrotransposons conserve this unusual feature demonstrates evolutionary pressure and suggests functionality [17,20]. Interestingly, the discovery of the antisense transcription in human telomeres, Telomeric Repeat-Containing RNA (TERRA), draws an additional common feature between these two kinds of chromosome ends [26].

#### 2.1.2. The Unusual Length of the 3′UTRs and Its Bias Composition

Besides bearing the promoter, there are to date, no more indications of functionality for the long 3′UTR of the HTT elements. Nevertheless, it is not unreasonable to suggest that the actual sequence per se, might be important. One possibility is the establishment of telomere chromatin. Interestingly, the DNA sequence of the entire telomere retrotransposons has a strong sequence bias, as the strand that runs 5′ to 3′ towards the centromere is extremely G-poor, resembling the same strand bias shown by telomerase repeats [9]. Maybe because this composition bias is important, we should mention that comparisons at the DNA and amino acid levels among the orthologues of the telomere retrotransposons showed a higher conservation at the DNA than at the amino acid level for most of the length of the telomeric retrotransposon [20].

#### 2.1.3. Coding Capacities of the *HTT* Elements

The level of conservation of the genes encoded by the telomere retrotransposons, *HeT-A gag*, *TART gag* and *pol*, suggests the existence of negative selective pressure [20]. Therefore, the proteins encoded by *HeT-A* and *TART* are likely necessary for their transposition and, as a consequence, for telomere elongation.

**Figure 2:**
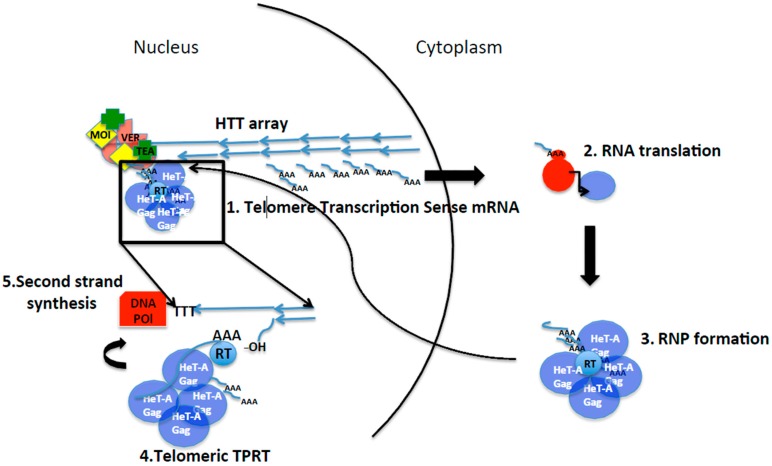
Life cycle of the telomeric retrotransposons in Drosophila. Black lines represent: division between nucleus and cytoplasm (curved), steps of the life cycle (arrows) and zoom in of the TPRT at the end of the chromosome (square). Small blue lines represent *HeT-A* mRNA and transcription from the telomeres. AAA poly A tail. For simplicity only *HeT-A* is represented. Blue arrows represent the elements at the *HTT* array, the telomeres in Drosophila. HeT-A Gag protein drawn as purple spheres. Red circles represent the ribosome and the red rectangle represents host DNA polymerase for second strand synthesis. 3′ hydroxyl group that primes the reverse transcription reaction is indicated. RT, reverse transcriptase (of still of unknown source). Moi, Tea, and Ver (MTV) are part of the capping complex that binds the ssDNA strand at the telomeres MTV, and is involved in formation of the spheres of HeT-A Gag containing *HeT-A* mRNA at the moment of telomere replication and elongation. See text for detail description of each step.

**Gag or Orfp1:** Previous studies have shown that the Gag protein of *HeT-A* is essential for telomere targeting of the telomere ribonucleoprotein (RNP) [27,28]. In contrast, despite entering the nucleus with high efficiency, the *TART* Gag protein does not localize to the telomeres on its own and instead requires *HeT-A* Gag [27,28,29]. Different works on the HeT-A Gag protein have demonstrated its involvement in the targeting mechanism of *HeT-A* and probably the other *HTT* mRNAs towards the end of the chromosome [14,30] (Figure 2).

The HeT-A Gag protein has also been found to establish interactions with chromatin-related proteins, such as Z4 and Nap-1, with consequences for telomere stability [15,31].

**Pol:** In addition, reverse transcription of the two elements at the end of the chromosome requires the enzymatic activities of a Pol protein. Both *TART* and *TAHRE* encode for a Pol protein composed of two different domains, an endonuclease (EN) of the Apurinic/Apyrimidinic (APE) class [32], and a reverse transcriptase (RT) (see Figure 1A).

Experimental evidences from recovered telomere ends where newly transposed elements have been detected [33], suggest that the *HTT* elements should transpose onto the end of the telomere with no need for DNA nicking. This scenario opens the question regarding the presence of a highly-conserved endonuclease activity in the Pol protein of both *TART* and *TAHRE* [8,20]. Alternative scenarios should be considered for reconciliation of these two pieces of evidence.

Because *HeT-A* is a non-autonomous element lacking the *pol* gene, two options have been put forward to explain its successful transposition. (1) Although there is not yet any biochemical demonstration of the involvement of the Pol gene from *TART*, the simultaneous presence of both, *HeT-A* and *TART* in all Drosophila genomes investigated to date makes their enzymatic collaboration the most parsimonious explanation [24,29,34,35,36]; (2) On the other hand, the *TAHRE* element combines the presence of a Gag protein, which highly resembles the *HeT-A* Gag protein, and an apparently functional Pol protein [8]. This combination would make *TAHRE* the best fitted of the three telomeric retrotransposons to replenish the telomere end. Nevertheless, only a few copies of the *TAHRE* element have been found in a few *Drosophila melanogaster* strains [37]. This scenario indicates that *TAHRE* transpositions are occasional and therefore cannot be considered a reliable source for telomere elongation. Because *HeT-A* and *TAHRE* share some unusual features it has been suggested that *HeT-A* might have evolved from *TAHRE* [37]. Nevertheless, the origin of *HeT-A* remains unclear.

Finally, it is worth mentioning that the main players of terminal transposition in Drosophila, *HeT-A* and *TART*, have been cloned in several species spanning 120 million years (MY) of genetic distance [35,36], revealing that although also committed to the essential function of telomere replication, are far from being static, and while maintaining their basic structures, allow their sequence to change rapidly, evolving faster than euchromatic genes and other retrotransposons [20]. As an example, we conducted a detailed study on *HeT-A* variability in different wild type and mutant backgrounds and demonstrated that at least nine different subfamilies of *HeT-A* were actively contributing to telomere extension in *D. melanogaster* [25]. Interestingly, other components of telomere biology in Drosophila, as for example those proteins involved in telomere capping function (end protection), are also fast evolving genes [38,39,40]). Although not linked, these two phenomena suggest that telomeres in Drosophila might be under an evolutionary pressure to change more rapidly than in other species.

### 2.2. Telomere Transposition and Life Cycle

#### Terminal Target Primed Reverse Transcription (TPRT)

**Figure 3:**
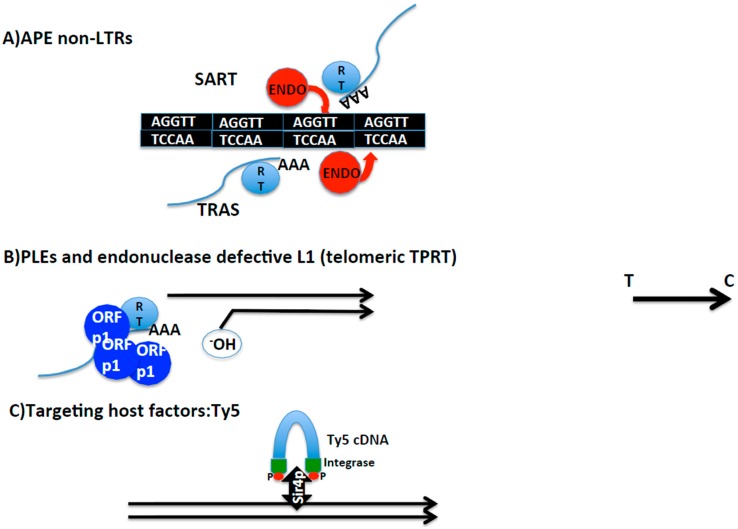
Schematic representation of different mechanisms of telomere integration. (**A**) Non-LTR retrotransposons containing Apurinic/Apyrimidinic (APE) endonucleases. *SART* and *TRAS* telomeric retrotransposons from *Bombyx mori.* Strand and nucleotide specificity of their respective endonucleases indicated. Telomere repeats in black. Blue lines: mRNA of *SART* and *TRAS*. RT indicates reverse transcriptase, and AAA poly A tail. (**B**) Penelope-like elements and endonuclease defective LINE L1 elements. Representation of telomeres by black lines. 3′ hydroxyl group that primes the reverse transcription reaction is indicated. Blue spheres indicate Orfp1 with structural properties, and RT, reverse transcriptase. Blue line represents retrotransposon mRNA (figuring PLE or L1) and AAA poly A tail. (**C**) Targeting host factors; Ty5 as an example. In blue, transposition intermediate of the LTR yeast retrotransposon Ty5. Integrase protein in green at the ends of the intermediate with red dots representing phosphorylation. Black and thick double arrow, Sir4P, the chromatin host factor that targets Ty5 towards subtelomeres. Black arrow on the right side of the figure indicates direction from telomere towards centromere applying to the entire figure.

*HeT-A*, *TART*, and *TAHRE* are non-LTR retrotransposons whose mechanism of transposition has been well studied and demonstrated for some of their counterparts, such as the mammalian Line L1, or the R1 and R2 of *D. melanogaster* [41,42,43]. In brief, canonical Target Primed Reverse Transcription (TPRT) would proceed as described in Figure 3 [44,45]. Non-LTR retrotransposons, once they are transcribed, their mRNAs are poly-adenylated and protected to travel to the cytoplasm where they will be used as template to produce their own proteins. A *cis*-preference interaction between proteins and mRNA has been demonstrated for L1 elements, ensuring in that way that the newly inserted copies are capable of transposition [46]. A RNP composed of translated Orf1 and Pol proteins together with the mRNA, will enter the nucleus following different strategies depending on each non-LTR retrotransposon (Figure 2). Once inside the nucleus, TPRT will take place [44,45]. In TPRT, the mRNA intermediate is directly reverse transcribed onto an internal nick in the chromosome produced by the retrotransposon endonuclease (see Figure 3). The reverse transcriptase of the element uses the 3′ hydroxyl group in the DNA produced by the endonuclease cut and the poly A to prime synthesis of the first DNA strand, beginning with the 3′ of the element mRNA. It is still unclear if the second strand of DNA is synthesized by the same reverse transcriptase or by the host DNA polymerases.

Telomeric TPRT in Drosophila is characterized by some slight variations. For example, as we have already seen in the previous section, *HeT-A* does not encode an RT, therefore at least one of the protein-mRNA interactions does not show a *cis*-reference. Another variation refers to the fact that apparently, because in the case of the telomeric transposons transposition should take place at the end, there is no need for nicking the DNA. Nevertheless, the signs of negative selection detected in the endonuclease domain of the *TART* element, and the conservation of those amino acids involved in endonuclease activity, strongly suggest the involvement of this activity for telomere transposition [20]. The easiest explanation for this contradiction could be that a nick consisting only of a few nucleotides would still be needed to start the reaction of reverse transcription. An alternative, less probable explanation studying the cloned elements from the telomeres would be the occasional internal insertion of the *HTT* elements in the telomeres, for which a nick would be essential. Because, as mentioned before, newly attached elements have been recovered in several experiments [33], a combination of both possibilities might be the most reasonable explanation. See also sections ahead where examples of telomere integrations in other organisms are explained.

Summarizing, telomere elongation in Drosophila is mainly achieved by successive transpositions of the *HTT* elements onto the end of the chromosome. *HeT-A*, *TART*, and *TAHRE* form long arrays always oriented in a head-to-tail direction with the poly A oriented towards the centromere [9,47].

### 2.3. Telomere Targeting

The first insights on how the telomere retrotransposons were being targeted to the telomeres came from in vitro studies of the cellular localization of *HTT* Gag proteins. The Gag protein of *HeT-A* is the only of the three telomeric Gags with the ability to localize at the telomeres (Figure 2) [28,29]. The telomere targeting of the HeT-A Gag protein has also been conserved across species [29]. *TART* and *TAHRE* depend on *HeT-A* for telomere targeting. Later, it was discovered that the HeT-A Gag protein formed aspheric structures that contained *HeT-A* RNA. Because the size of the Gag spheres at the telomeres increase in a mutant background known to have increased *HeT-A* transcription, the *Su(var2-5)* mutant [48], the involvement of the *HeT-A* mRNA in telomere targeting was suggested [30]. Importantly these aspheric *HeT-A* Gag-mRNA complexes were only seen at the telomeres, indicating once more that the telomere targeting of the telomere transposons is highly specific. It is worth mentioning that this feature is key for the stability of the retrotransposon telomeres. If internal insertion of the telomere retrotransposons would be possible, homologous recombination between copies at the telomeres with copies inside the chromosome arms could occur with fatal consequences.

A second key discovery about telomere targeting in Drosophila was the involvement of the Verrocchio (Ver) protein in the formation of the Gag spheres [30]. Ver was at the time defined as a protein belonging to the capping complex [39,49]. Later studies have redefined Ver as essential component of the Moi-Tea-Ver (MTV) complex, the ssDNA binding complex essential for telomere capping function in Drosophila [14,50]. This discovery couples the protection of the end of the chromosome with the targeting of the *HeT-A* transposition machinery to the telomeres making possible the connection between the two defining functions of a telomere, end elongation and protection [51,52].

Finally, we should consider that because the telomere retrotransposons maintain their characteristics as TEs, telomere-targeting in *Drosophila* might be under different layers of control. As mentioned before, telomeres are a genomic compartment with specific chromatin characteristics and it is therefore reasonable to consider chromatin characteristics at telomeres to be one of the factors that might influence retrotransposon insertion. Indeed, we and others have found several interactions between telomere retrotransposon proteins and chromatin complexes [12,14,15,31,53,54].

### 2.4. Other Cases of Telomere Transposition and Integration

Although Drosophila is the only organism to date where the transposition of retrotransposons has been tamed to occur when telomere elongation is needed and is coupled with telomere replication, telomere transposition has also been described in other organisms.

Telomere integration in other organisms besides Drosophila can follow different strategies (Figure 3).

#### 2.4.1. Transposition Inside Telomere Repeats

Interestingly, there are two more insect species, *Bombyx mori* and *Tribolium castaneum*, that contain non-LTR retrotransposons in their telomeres. In this case, opposite to Drosophila, telomerase is still found in their genomes. *SARTBm*, *SARTTc*, and *TRASBm* and *TRASTc* insert inside the telomeric repeats of each species guided by the specificity of their endonuclease domain [45,55] (Figure 3). Because *SART* and *TRAS* from both species have been phylogenetically related, I will generically refer to them by *SART* and *TRAS*. *SART* and *TRAS* are non-LTR retrotransposons that encode for an endonuclease domain of the APE class. APE endonucleases have been shown to have target specificity. The case of *SART* and *TRAS* endonucleases is particularly illustrative since their respective endonucleases show specificity for the reverse target site, giving the reason for their palindromic name [32,45,56] (see Figure 3).

Interestingly, in these two organisms the reverse transcriptase domain of telomerase, TERT, has lost some of the important motifs for effective RT processing [57]. It is possible, therefore, that evolution may favor the transposition of *SART* and *TRAS* inside the telomere repeats, buffering in this way the length of the telomere sequence [55]. *SART* and *TRAS* in *B. mori* make up more than the 3% of the genome concentrated in more than a 1000 copies [45].

An additional piece of information applying to the *TRAS* elements is the presence of a Myb domain in the Pol. Myb domains have been found in several telomeric proteins and are important for their interaction with the telomeric sequence, suggesting that in the case of *TRAS* the Myb domain may also have play a role in the specificity of its telomere integration [45].

*Girardia lambia* is a parasitic protozoan with a polyploid genome that also contains non-LTR retrotransposons inserted at its telomeres. *GilT*, *GilM*, and *GilD*, are found specifically inserted between the subtelomeric regions and the telomere repeats in this genome [58]. The elements are found organized in head-to-tail arrays always in the same direction, suggesting insertion by transposition. Interestingly, some of the elements show 5′ truncations indicative of having been at the end of the chromosome and suffering terminal erosion. As is the case of the telomeric transposons in Drosophila, *GilT, GilM*, and *GilD* have unusually long 3′UTRs for being non-LTR retrotransposons. It is not clear how the specificity of integration in this case is achieved.

Other cases of non-LTR retrotransposons inserted at telomeres much less studied are; the *Zepp1* elements present in all telomeres from the green algae *Chlorella vulgaris*, the *MoTeR* elements that show specificity for the telomere repeats in the fungus *Magnaporthe oryzae*, and the recently found *Tx1-1_ACar* also in the telomeric repeats of the anole lizard revised in [45].

#### 2.4.2. Targeting of Telomere Chromatin

A different way to integrate at a specific genomic location is by interacting with a host factor. There are many examples of mobile elements and viruses that make contact with chromatin complexes or specific modifications of histones to be tethered to silenced or actively transcribed regions of the genome [59]. Among these examples, one that has been remarkably well studied is the case of the LTR retrotransposon Ty5, which inserts in the subtelomeric regions of *Saccharomyces cerevisiae* [60,61]. Ty5 integrase interacts with Silencing Information 4 protein (Sir4p). The interaction with Sirp4 targets the insertion to subtelomeres and to the silent mating locus. The interaction between the Ty5 integrase and Sir4p is dependent on the phosphorylation of the Ty5 integrase, and this event occurs dependent on the stress conditions. This scenario reminds one of Barbara McClintock’s thesis on mobile elements, being a reservoir to be explored when rewiring of genetic networks would be desired, as for example in the event of a change in the environment [62].

For more examples of specific targeting of mobile elements and viral integration in eukaryotes elsewhere than telomeres, see a recent review from Pascal Lesage [59].

As mentioned earlier, although not directly involved in telomere targeting, the HeT-A Gag protein was also found to interact with chromatin components in Drosophila such as the Z4/putzig protein. The disruption of this interaction has fatal consequences for telomere stability [15].

#### 2.4.3. Occasional End-Transposition

The third mechanism that has been involved in telomere targeting gives clues on the evolution and functional connections between mobile elements and telomeres. There are mainly two examples in this third class of telomere integration specificity, the endonuclease defective elements and the Penelope-like elements (PLE). PLE are a new class of retroelements described a few years ago with some features shared with Group I of introns [63]. Canonical PLEs are defined by being three to four kilobases in length, and framed by terminal repeats that can be either direct or inverted, called pLTRs [56]. The first PLEs described contained an endonuclease domain, the GIY-YIG, which was different from the one seen in non-LTR and LTR retrotransposons, and in common with Group I introns [64].

Following on, *Athena*, a new family of PLEs in the bdelloid rotifers *Philodina roseola* and *Adineta vaga*, which lacked an endonuclease domain, was found to transpose at the telomeres [65]. Most of the copies contained a short stretch of telomeric repeats at their 3′ end, indicating that at the time of transposition of these PLEs, the telomere was probably uncapped and the downstream genes were exposed. Supporting this hypothesis, some of the *Athena* elements were 5′ truncated which would agree with their position, at least momentarily, at the end of the chromosome. Interestingly, phylogenetic analysis of the RT from *Athena* elements, revealed this RT as the closest relative to eukaryote telomerases.

The lack of an endonuclease domain in the *Athena* elements coincides with the discovery that Line L1 elements defective in their endonuclease activity are able to insert at the end of the chromosome when the murine cell lines are defective in the non-homologous end joining (NHEJ) pathway [66].

During the course of writing this review, a new kind of retrotransposon with telomere specificity has been described [56], the *Terminons*. *Terminons* are defined as long units that can expand up to 40 kb in length, contain RTs of the *Athena* family, and endonucleases of the GIY-YIG class, and are found in bdelloid rotifers. *Terminons* can also acquire host Orfs that accumulate in their genome in an oriented manner and could contribute to their life cycle. As is the case for the other retrotransposons suspected to be at the end of the chromosome, *Terminons* are also often 5′ truncated. Because of their large genomes and their capacity to accept several Orfs, *Terminons* might hide important connections to the viral world.

Indeed, several herpesviruses are able to integrate their genome at the telomeres in humans [67]. Integration happens during the latency phase and in some cases reaches through germ cells, which will allow not only their presence in the host for life, but also being vertically transmitted to the next generation [68,69,70].

As a part of a special issue dedicated to telomere integration with more specialized authors in viral integration, I will not revise here the specific mechanisms that have been recently elucidated [67], but I would like to summarize some components that have been shown to be of importance for such integration. The viral genomes with telomere specificity are flanked by direct repeats, telomeric repeats (TMR) and perfect telomeric repeats (pTMR), which at least in one side are highly identical to human telomerase repeats [70]. This feature has also been found in the recently described retrotransposons, *Terminons* (see above). Some of the viral genomes encode for proteins that are involved with the telomerase mechanism. The Marek’s disease virus (MDV) encodes a Telomerase RNA gene, *vTR*, whose expression enhances telomerase activity, suggesting an important connection with telomere biology. Other genes encoded by viral genomes that integrate at telomeres are exonucleases, UL12, UL29, and recombinases U94 [67]. Finally, several host factors that could be involved in different integration mechanisms are Rad51 and DMC1 or other host components involved in recombination [67].

It is likely that not just one, but several mechanisms are involved in viral telomere integration. In this review, we have just seen the variety of mechanism for retrotransposon integration at telomeres. Therefore, one could put forward several non-mutually exclusive possibilities underlying the different mechanisms. As an example of the most intuitive ones: interplay between viral and host factors, similarities in sequences to be reverse transcribed by the telomerase machinery, interaction with the recombination machinery, and interaction with components of the telomere protection complex (“sheltering”). Finally, it is very likely that co-evolution of viral and host genomes plays an important role to understand all the possible mechanisms that are, or could be in the future, involved in this fascinating interaction.

## 3. Similarities between the Telomeric Retrotransposons in Drosophila and Telomerase

While the *HTT* retrotransposons might seem very different from telomerase repeats, in both cases they are copied by reverse transcription of an RNA template (TR for telomerase organisms and *HeT-A* mRNA in Drosophila), increasing their copy number at the end of the chromosome, buffering in this way the receding telomere [5,47]. In both systems the basic RNP needs the assistance of additional proteins for processivity, telomere targeting, and regulation [9,71].

An additional similarity of both mechanisms comes from having in mind, once more, that although *HeT-A* is the most abundant of the three elements in the *HTT* array, it lacks the enzymatic activities needed to transpose. Therefore, as in the case of telomerase telomeres, the RT activity (TERT in telomerase telomeres and RT likely from *TART* or *TAHRE* in Drosophila) and the RNA template (TR in Telomerase telomeres and *HeT-A* mRNA in Drosophila) come from different genetic units [3,6].

Of special importance for this section are two discoveries done in the last years by Yikang Rong’s laboratory. First, this laboratory demonstrated that, as is the case for the organisms bearing telomerase, telomere replication and elongation were also coupled in Drosophila [72,73,74]. It was shown that the HeT-A Gag protein (named Orfp1 in this paper [30]) was mainly expressed in the early S-phase, when telomere replication had been described. The HeT-A Gag protein was interacting with the *HeT-A* mRNA with the assistance of the Ver protein (see below). This complex of HeT-A Gag, mRNA, and Ver forms spherical structures at the telomeres, which then participate in telomere elongation [30,74,75].

Secondly, it was demonstrated that Ver, known to bind ssDNA in Drosophila, could be considered the equivalent of a protein in yeast that had also been shown to bind ss-DNA at the telomeres, the Stn-1 protein [76]. The reason behind this equivalence was that both Stn-1 and Ver are involved in telomere capping in their respective organisms [39,76], likely controlling also the access of telomerase or *HeT-A* intermediates of transposition in their respective organisms. This new parallelism of function between these two proteins draws an additional mechanistic link between telomerase and retrotransposon telomeres.

Finally, it has also been reported that mutations in Ars2, a protein first described as a regulator of TERRA transcription in telomerase organisms, produce an excess in *HeT-A* transcription in the germ line causing mitotic defects in the embryos [77]. Importantly, mutations in Ars2 in telomerase organisms also cause genetic instability [78].

In summary, as more functional and mechanistic features of the telomeres of Drosophila are being revealed, more similarities are drawn with the telomerase mechanism. Nevertheless, the main difference between both systems is in the inherent specific entity of the telomere repeats as retroelements in the case of Drosophila. This fact implies an additional difference between the two mechanisms. In the case of Drosophila, the machinery involved in telomere maintenance is embedded in the actual telomere. Telomeres have their own functional and chromatin particularities, adding an extra layer of complexity to the homeostasis of Drosophila telomeres.

## 4. Evolutionary Considerations; Telomeres and Mobile Elements

PLE elements do not show an LTR or non-LTR structure, are quite diverse, and have some features reminiscent of Group I introns. The phylogenetic analysis of the RT domains of these newly discovered elements suggested that PLE RTs were the most ancient of all retroelements [63,65,79]. PLEs with *Athena* RTs cluster together with eukaryote telomerases and place *Athena* RTs as the ancestor RT for TERT and the RTs for non-LTR retrotransposons [56,80].

Most RT domains work in coordination with extra domains with additional enzymatic activities, as endonucleases, integrases, recombinases or GIY-YIG nickases [63]. The RT domain might have been initially alone and the progressive addition of the other activities likely widened the possibilities of insertion around the genome, freeing TEs from having to rely on the repair of double strand breaks, replication forks, or jumping at the end of the chromosome in order to transpose. Once specialized retrotransposons increased their efficiency and their chance of transposition, they became selfish elements, spreading their copies throughout the genome [80]. In favor of this hypothesis, lies the fact that different cases of endonuclease-defective retrotransposons manage to insert at the end of the chromosome. Actually, connections towards this relationship also exist coming from the other direction, because telomerase has been shown to be able to occasionally reverse transcribe telomere repeats at internal genomic locations resembling a “transposition” event of telomerase repeats [81]. Finally, the very recent discovery of *Terminons* also supports this chain of events. In the large *Terminons* units, the RT activity is always found, while the GIY-YIG endonuclease and the rest of extra co-opted Orfs can be found in some of but not all *Terminons* units [56].

## 5. Conclusions

As we have just seen, there are different strategies to get inserted at the telomeres, which respond to different factors of the inserted sequence or of the telomere. In any case, detected telomere integrations are the result of successful integration and selection. We have seen in this review that this balance has resulted in many different examples from both recent integrations as well as integrated events along evolution, suggesting that telomeres are able to accept exogenous sequences and still perform efficiently enough to prevent genome instability.

Work from telomerase exceptions has been crucial to elucidate one of the characteristics that might explain why telomeres may be more plastic than previously thought. Extensive work in Drosophila has shown that the actual sequence at the telomeres is not important for protecting the end of the chromosome and avoiding genome instability. The capping complex is able to associate independently of the presence of *HeT-A* elements at a particular telomere. Nevertheless, in all these studies, *HeT-A* elements are still present at some telomeres and their contribution in *cis* to, for example, telomere targeting, cannot be ruled out. Interestingly, we have reviewed several cases that indicate that although a specific sequence is not necessary, there are features of nucleotide composition whose conservation suggest certain requirements for being at the telomeres. Examples such as the strand bias composition for telomerase, the telomeric retrotransposons in Drosophila, or the incorporation of telomere repeat sequences in their genetic units for different Herpesvirus [69], or GilM, GilT, and GilD, [63] favor this hypothesis.

Along this review we have also seen how different classes of transposons are able to jump at the end of the chromosome when this is accessible and the particular copy lacks the enzymatic activities needed to nick the DNA. It is likely that evolution has played with this possibility to buffer and protect the ends of the chromosomes in events of loss of the telomerase or a down regulation of its processivity.

Finally, the newly described *Terminons* demonstrate that as more genomes are investigated, new examples of non-telomerase sequences might be found at the end of the chromosomes, and more connections between TEs and telomeres might be described. Here we have shown the basis for this mechanistic relationship which lies under a common evolutionary origin. Treasuring the exceptions and understanding the detailed mechanisms in different pairs of telomeres-TE will help to understand the nature of the newly integrated viral genomes in human telomeres. In view of the many different strategies that their cousins, retroelements, have worked out how to be inserted at this particular chromosomal territory, it would be naive to think that there is a universal mechanism for viral integration at telomeres. The plasticity that telomeres seem to have for accepting different sequences is probably one of the factors that allows this phenomenon to occur and persist.

Drosophila telomeres have been the telomerase exception most studied to date, and keep being an open door to perform studies that will undoubtedly help to understand the complicated and fascinating relationship between genomes and transposable elements. Importantly, the telomeric retrotransposons in Drosophila are the only ones so far demonstrated to be perfectly tamed to perform the telomeric function when needed, and being robustly integrated with telomere biology in this species for more than 120 MY.
